# Supplementary material for: Blue Vane and Pan Traps Are More Effective for Profiling Multiple Facets of Bee Diversity in Subtropical Forests
Source: Insects. 2024 Nov 20;15(11):909. doi: 10.3390/insects15110909 (PMC11594821; doi:10.3390/insects15110909)
Supplement: Supplementary file 1 [file insects-15-00909-s001.zip › insects-3258943-supplementary.pdf]

ADDITIONAL FILES
SUPPORTING INFORMATION

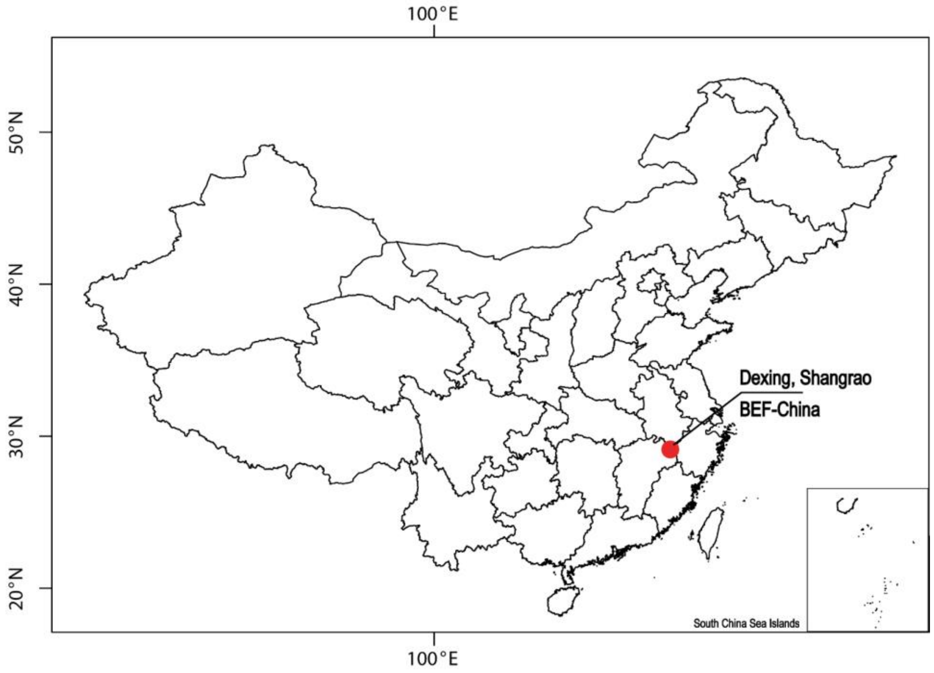

Figure S1. The location of BEF-China experiment.

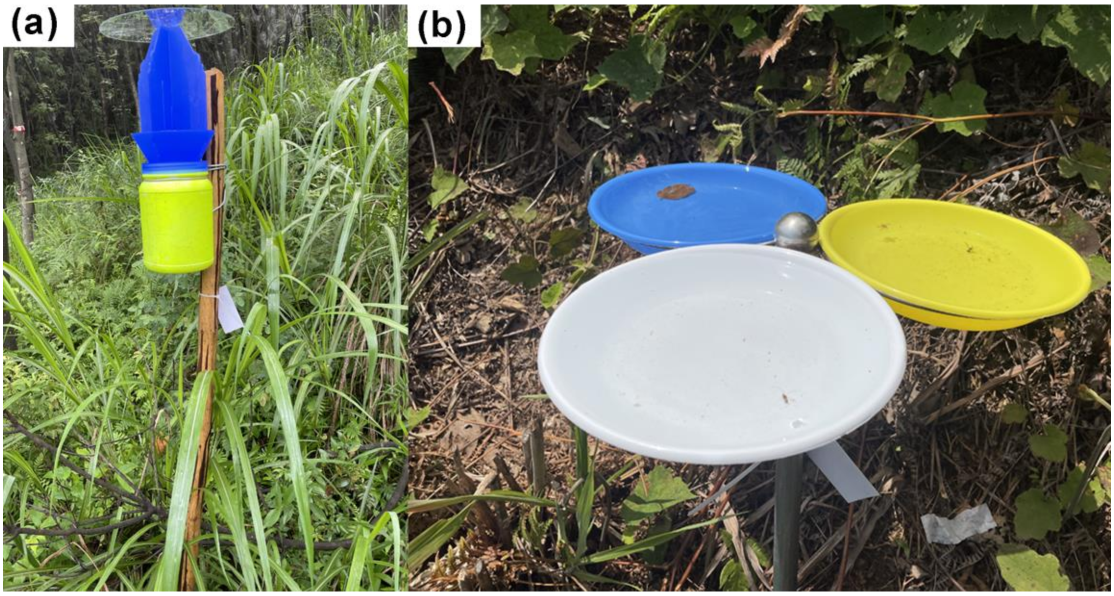

Figure S2. (**a**) Blue vane traps were hung from wooden sticks at a height of approximately 1 m, and (**b**) three-colored pan traps were positioned by metal brackets at a height of 0.5 m.

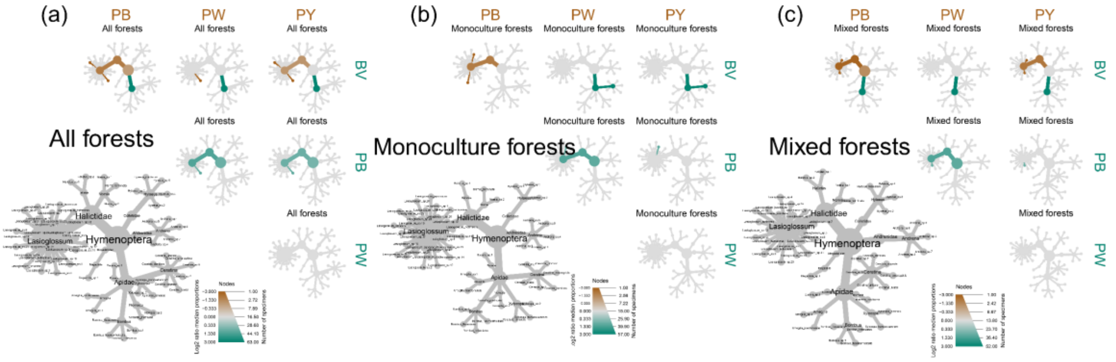

Figure S3. Pairwise comparisons of bee abundance and composition among (**a**) all forests (monoculture and mixed plots), (**b**) monoculture forests and, (**c**) mixed forests. The grey trees of the lower left show complete taxonomies. Smaller trees represent pairwise comparisons between trap types. Branches in brown denote higher abundance of those of the column, and green indicate higher abundance across trap types shown on rows. The node colors represent the difference among compared trap types evaluated by log2 ratio of median proportions and the node size represent the number of bee species at each taxonomic level. Abbreviations: PB, blue pan trap; PW, white pan trap; PY, yellow pan trap; and BV, blue vane trap.

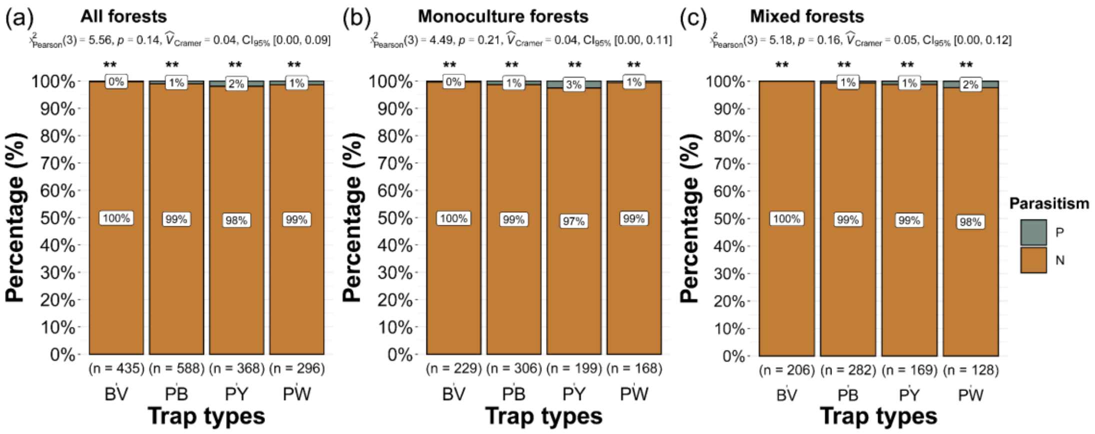

Figure S4. Parasitism distribution of bees captured by four trap types in (**a**) all forests (monoculture and mixed plots), (**b**) monoculture forests, and (**c**) mixed forests. The bar charts present the distribution of parasitic behavior. ** denotes $p < 0.01$. Abbreviations: P, cleptoparasitic; N, non-parasitic; PB, blue pan trap; PW, white pan trap; PY, yellow pan trap; and BV, blue vane trap.

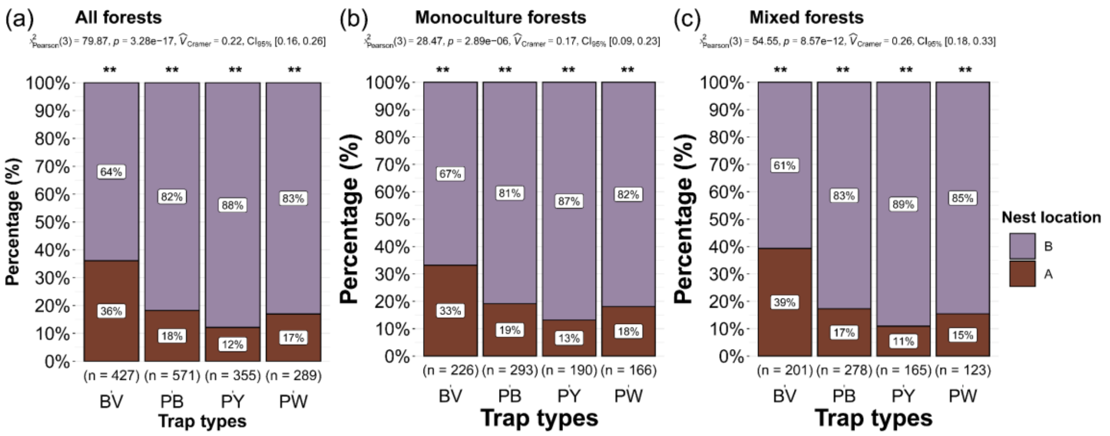

Figure S5. Nesting location distribution of bees captured by four trap types in (**a**) all forests (monoculture and mixed plots), (**b**) monoculture forests, and (**c**) mixed forests. The bar charts present the distribution of nesting locations. ** denotes $p < 0.01$. Abbreviations: A, nesting above-ground; B, nesting below-ground; PB, blue pan trap; PW, white pan trap; PY, yellow pan trap; and BV, blue vane trap.

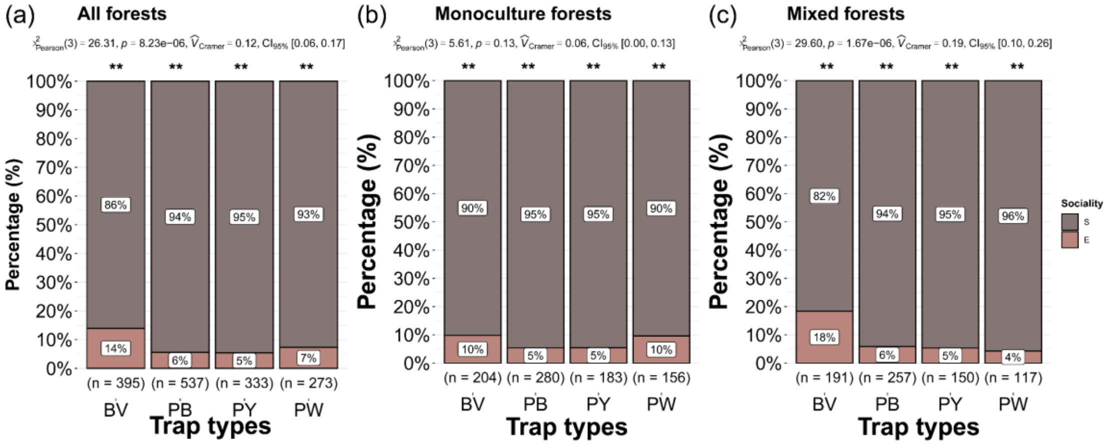

Figure S6. Sociality of bees captured by four trap types in (**a**) all forests (monoculture and mixed plots), (**b**) monoculture forests, and (**c**) mixed forests. The bar charts present the distribution of sociality. ** denotes $p < 0.01$. Abbreviations: S, solitary; E, eusocial; PB, blue pan trap; PW, white pan trap; PY, yellow pan trap; and BV, blue vane trap.

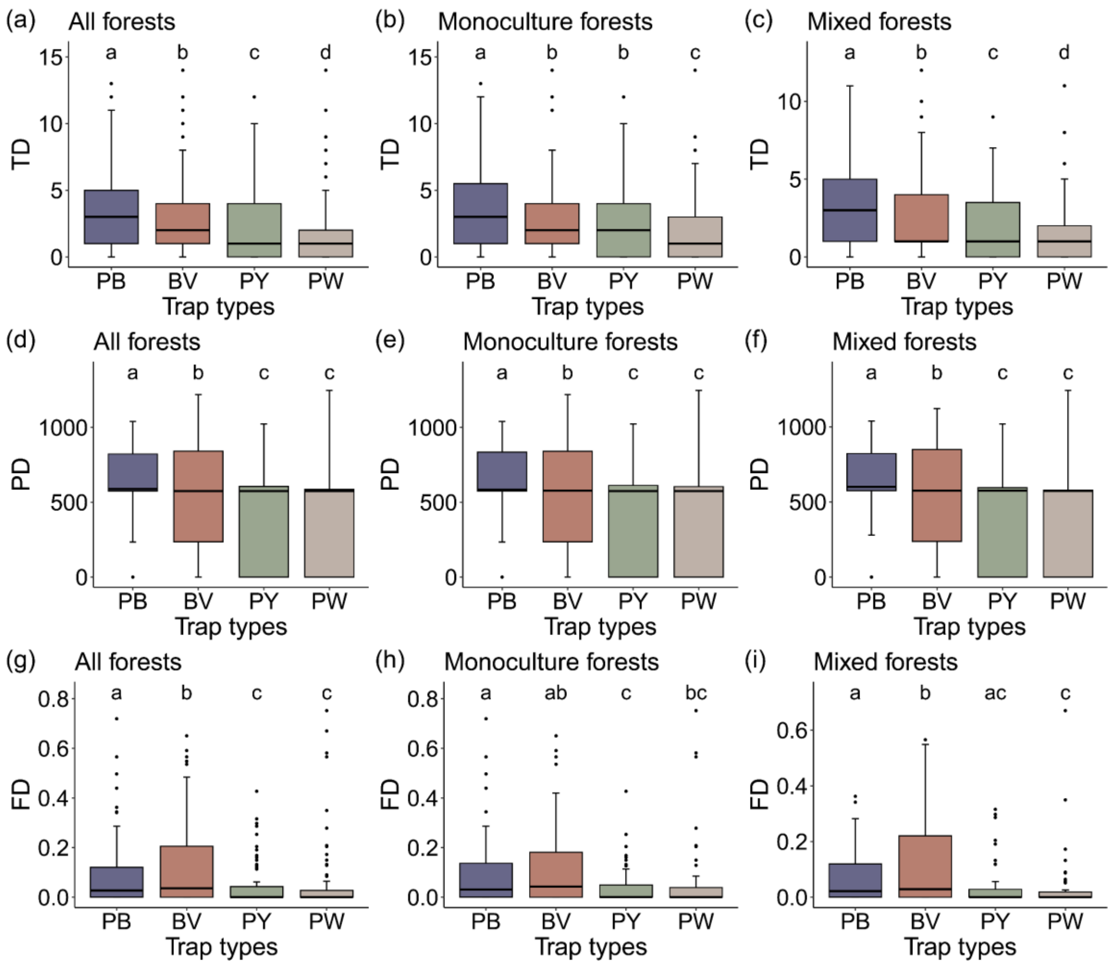

Figure S7. Comparisons of three facets of bee diversity across four trap types in all forests (monoculture and mixed plots, 1st column), monoculture forests (2nd column), and mixed forests (3rd column). Diversity was estimated for (**a**)–(**c**) TD; (**d**)–(**f**) PD, and (**g**)–(**i**) FD. Circles indicate outliers. Letters represent statistical differences according to one-tailed pairwise Wilcoxon test for non-parametric data, with groupings denoted by shared letters ($p > 0.05$). p values were adjusted by the Benjamini-Hochberg (BH) method. Abbreviations: TD, species richness; PD, Faith's phylogenetic diversity; FD, functional richness; PB, blue pan trap; PW, white pan trap; PY, yellow pan trap; and BV, blue vane trap.

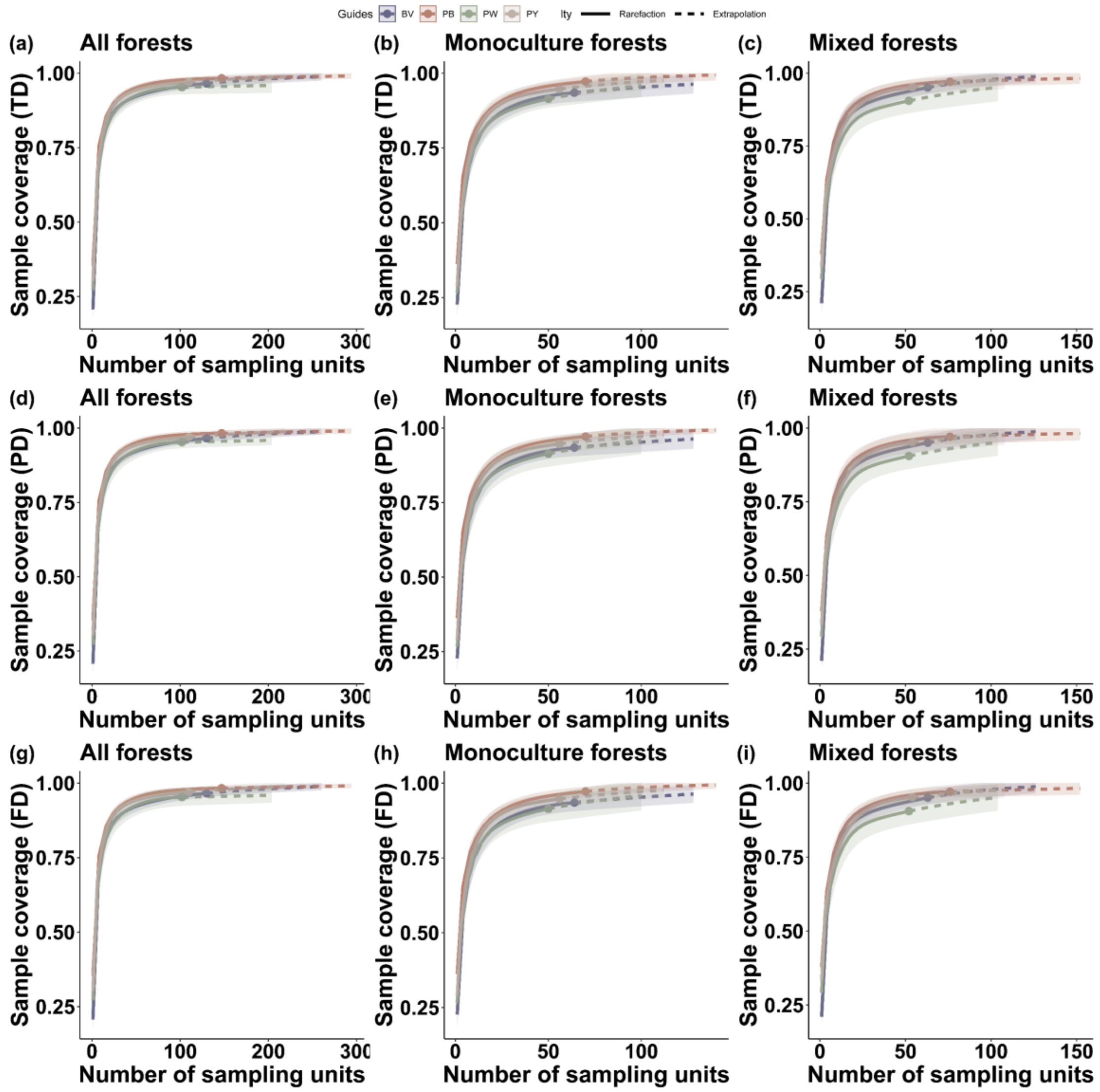

Figure S8. Coverage-based rarefaction (solid lines) and extrapolation (dotted lines) of bees captured by four trap types, with 95% unconditional confidence intervals (shading) in all forests (monoculture and mixed plots, 1st column), monoculture forests (2nd column), and mixed forests (3rd column). Diversity was estimated for (**a**)–(**c**) TD; (**d**)–(**f**) PD, and (**g**) –(**i**) FD. Abbreviations: TD, taxonomic diversity; PD, phylogenetic diversity; FD, functional diversity; PB, blue pan trap; PW, white pan trap; PY, yellow pan trap; and BV, blue vane trap.

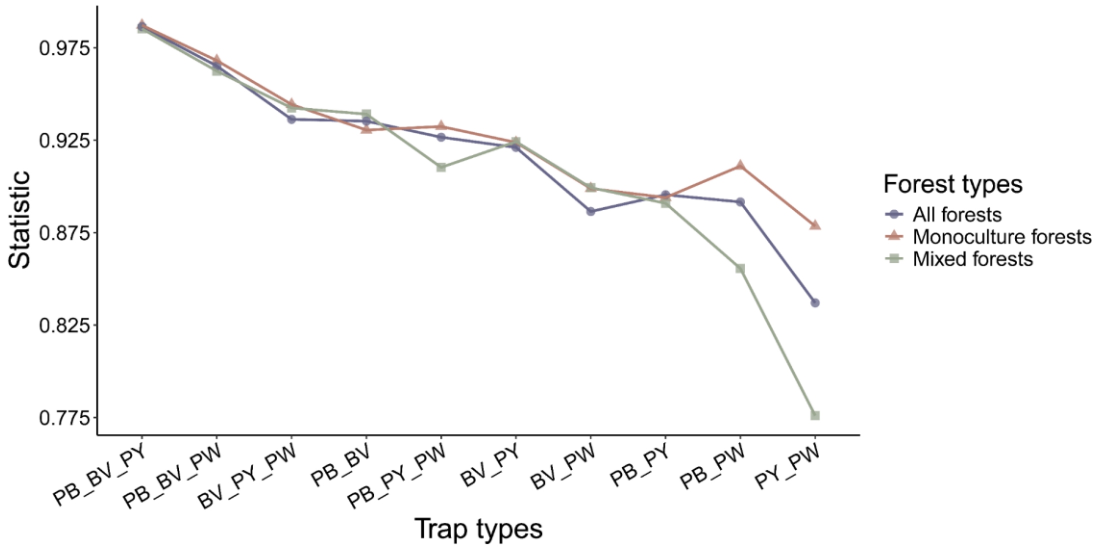

Figure S9. Mantel correlation coefficients (r_M) between the faunal similarity matrix based on different combinations and the complete survey. Abbreviations: PB, blue pan trap; PW, white pan trap; PY, yellow pan trap; and BV, blue vane trap.

Table S1. Life-history trait descriptions.

Trait	states	Definition
Sociality	Eusocial	Division of labor, queens and workers are morphologically different (including eusocial and primitively eusocial)
	Social	Division of labor (including semi-social, sub-social, facultative social)
	Solitary	Lacking division of labor (including solitary and communal)
Nest location	Below	Nesting below ground
	Above	Nesting above ground
Parasitism	Non-parasitic	
	Cleptoparasitic	Females lay their eggs in nests of specific host species (parasitize solitary hosts)
	Socially parasitic	Parasitizing social hosts

Table S2. Taxonomic information for specimen collected under different forest types (tree diversity level).

	All	Monoculture	Mixed
**Andrenidae**	**19**	**13**	**6**
Andrena	**19**	**13**	**6**
Andrena sp.1	5	3	2
Andrena sp.4	2	1	1
Andrena sp.5	4	3	1
Andrena sp.6	2	1	1
Andrena sp.7	6	5	1
**Apidae**	**940**	**597**	**343**
Amegilla	**31**	**15**	**16**
Amegilla korotonensis Cockerell, 1911	29	14	15
Amegilla sp.1	2	1	1
Bombus	**79**	**27**	**52**
Bombus flavescens Smith, 1852	37	14	23
Bombus haemorrhoidalis Smith, 1852	20	6	14
Bombus sp.1	17	5	12
Bombus trifasciatus Smith, 1852	5	2	3
Ceratina	**777**	**525**	**252**
Ceratina cognata Smith, 1879	2	0	2
Ceratina dentipes Friese, 1914	13	9	4
Ceratina iwatai Yasumatsu, 1936	127	83	44
Ceratina smaragdula Fabricius, 1787	28	15	13
Ceratina sp.7	502	337	165
Ceratina sp.8	93	74	19
Ceratina unicolor Friese, 1911	12	7	5
Eucera	**16**	**11**	**5**
Eucera sp.1	16	11	5
Nomada	**19**	**11**	**8**
Nomada posthuman Blüthgen, 1949	18	10	8
Nomada sp.2	1	1	0
Xylocopa	**18**	**8**	**10**
Xylocopa sinensis Smith, 1854	13	6	7
Xylocopa sp.2	1	1	0
Xylocopa tranquebarorum Swederus, 1787	4	1	3
**Colletidae**	**45**	**19**	**26**
Hylaeus	**45**	**19**	**26**
Hylaeus insularum Yasumatsu & Hirashima, 1965	1	0	1
Hylaeus sp.2	29	11	18
Hylaeus sp.3	12	7	5
Hylaeus sp.4	2	0	2
Hylaeus sp.5	1	1	0
**Halictidae**	**2987**	**1601**	**1386**

Halictus	**7**	**4**	**3**
Halictus sp.1	6	4	2
Halictus sp.2	1	0	1
Lasioglossum	**2965**	**1590**	**1375**
Lasioglossum hoffmanni Strand, 1915	26	12	14
Lasioglossum japonicum Dalla Torre, 1896	36	21	15
Lasioglossum kansuense Blüthgen, 1934	112	78	34
Lasioglossum occidens Smith, 1873	183	111	72
Lasioglossum sp.1	1141	551	590
Lasioglossum sp.10	22	15	7
Lasioglossum sp.11	8	4	4
Lasioglossum sp.12	1	1	0
Lasioglossum sp.13	4	3	1
Lasioglossum sp.14	1	1	0
Lasioglossum sp.16	2	2	0
Lasioglossum sp.17	3	3	0
Lasioglossum sp.18	1	1	0
Lasioglossum sp.2	667	345	322
Lasioglossum sp.20	6	3	3
Lasioglossum sp.23	2	1	1
Lasioglossum sp.24	10	6	4
Lasioglossum sp.26	3	3	0
Lasioglossum sp.27	1	1	0
Lasioglossum sp.28	2	1	1
Lasioglossum sp.29	3	1	2
Lasioglossum sp.3	162	99	63
Lasioglossum sp.4	76	38	38
Lasioglossum sp.5	37	19	18
Lasioglossum sp.6	178	94	84
Lasioglossum sp.7	120	89	31
Lasioglossum sp.9	9	7	2
Lasioglossum subopacum Smith, 1853	149	80	69
Nomia	**15**	**7**	**8**
Nomia sp.1	1	0	1
Nomia sp.2	1	1	0
Nomia terminate Smith, 1876	13	6	7
**Megachilidae**	**2**	**0**	**2**
Megachile	**2**	**0**	**2**
Megachile sp.1	2	0	2
**In total**	**3993**	**2230**	**1763**

Column total gives the number of specimens assigned to taxon.

Table S3. Results of Shapiro-Wilk test of normality and Bartlett test of homogeneity of variances for comparisons of inter-tegular distance (ITD).

Forest types	Shapiro-Wilk test		Bartlett test		
	W	*p* value	K-squared	df	*p* value
All	0.65	**<0.001**	627.03	3	**<0.001**
Monoculture	0.64	**<0.001**	345.62	3	**<0.001**
Mixed	0.65	**<0.001**	298.55	3	**<0.001**

Significant results are indicated in bold.

Table S4. Results of Shapiro-Wilk test of normality and Bartlett test of homogeneity of variances for comparisons of taxonomic, phylogenetic and functional diversity.

Forest types	Diversity	Shapiro-Wilk test		Bartlett test		
		W	*p* value	K-squared	df	*p* value
All	Taxonomic	0.84	**<0.001**	10.69	3	**<0.05**
	Phylogenetic	0.88	**<0.001**	25.50	3	**<0.001**
	Functional	0.63	**<0.001**	55.68	3	**<0.001**
Monoculture	Taxonomic	0.84	**<0.001**	4.30	3	0.231
	Phylogenetic	0.88	**<0.001**	10.60	3	**<0.05**
	Functional	0.62	**<0.001**	31.06	3	**<0.001**
Mixed	Taxonomic	0.84	**<0.001**	8.73	3	**<0.05**
	Phylogenetic	0.87	**<0.001**	15.30	3	**<0.01**
	Functional	0.64	**<0.001**	30.52	3	**<0.001**

Significant results are indicated in bold.

Table S5. Comparisons of inter-tegular distance (ITD) across different forest types (tree diversity level) obtained from one-tailed pairwise Wilcoxon test.

**Group1**	**Group2**	**Counts1**	**Counts2**	**Statistic**	**Counts1**	**Counts2**	**Statistic**	**Counts1**	**Counts2**	**Statistic**
		**Taxonomic**			**Phylogenetic**			**Functional**		
BV	PB	435	588	**157870.50****	229	306	**44233.50****	206	282	**34933.50****
BV	PY	435	368	**102407.50****	229	199	**29500.00****	206	169	**21976.50****
BV	PW	435	296	**82428.50****	229	168	**24378.50****	206	128	**17049.00****
PB	BV	588	435	97909.50	306	229	25840.50	282	206	23158.50
PB	PY	588	368	112491.00	306	199	30983.00	282	169	25341.00
PB	PW	588	296	91104.50	306	168	25731.00	282	128	19947.5
PY	BV	368	435	57672.50	199	229	16071.00	169	206	12837.500
PY	PB	368	588	103893.00	199	306	29911.00	169	282	22317.00
PY	PW	368	296	54906.50	199	168	16395.00	169	128	11284.50
PW	BV	296	435	46331.50	168	229	14093.50	128	206	9319.00
PW	PB	296	588	82943.50	168	306	25677.00	128	282	16148.50
PW	PY	296	368	54021.50	168	199	17037.00	128	169	10347.50

Significant difference is indicated in bold;
** denotes $p < 0.01$; * denotes $p < 0.05$.

Table S6. Comparisons of three facets of bee diversity in monoculture forests obtained from one-tailed pairwise Wilcoxon test.

Group1	Group2	Counts1	Counts2	Statistic	Counts1	Counts2	Statistic	Counts1	Counts2	Statistic
		Taxonomic			Phylogenetic			Functional		
PB	BV	79	79	**1388.00****	79	79	**1722.00***	52	46	183.00
PB	PY	79	79	**1730.50****	79	79	**2157.00****	52	55	**506.00****
PB	PW	79	79	**2081.50****	79	79	**2212.00****	52	53	**399.00****
BV	PB	79	79	442.00	79	79	1053.00	46	52	282.00
BV	PY	79	79	991.00	79	79	**1541.50***	46	55	**286.00***
BV	PW	79	79	**1256.00****	79	79	**1553.50****	46	53	201.00
PY	PB	79	79	222.50	79	79	399.00	55	52	124.00
PY	BV	79	79	662.00	79	79	804.50	55	46	92.00
PY	PW	79	79	**953.00***	79	79	1088.50	55	53	173.00.
PW	PB	79	79	129.50	79	79	416.00	53	52	66.00
PW	BV	79	79	455.00	79	79	526.50	53	46	99.00
PW	PY	79	79	478.00	79	79	741.50	53	55	178.00

Significant difference is indicated in bold;
** denotes $p < 0.01$; * denotes $p < 0.05$.

Table S7. Comparisons of three facets of bee diversity in mixed forests obtained from one-tailed pairwise Wilcoxon test.

**Group1**	**Group2**	**Counts1**	**Counts2**	**Statistic**	**Counts1**	**Counts2**	**Statistic**	**Counts1**	**Counts2**	**Statistic**
		**Taxonomic**			**Phylogenetic**			**Functional**		
PB	BV	83	83	**1964.00****	83	83	**2057.00****	53	46	68.00
PB	PY	83	83	**2190.00****	83	83	**2616.50****	53	55	374.00
PB	PW	83	83	**2328.00****	83	83	**2357.00****	53	47	**300.00****
BV	PB	83	83	521.00	83	83	1103.00	46	53	**310.00****
BV	PY	83	83	**1329.50****	83	83	**1901.50****	46	55	**210.00****
BV	PW	83	83	**1576.50****	83	83	**1756.00****	46	47	**145.00****
PY	PB	83	83	225.00	83	83	386.50	55	53	187.00
PY	BV	83	83	750.50	83	83	799.50	55	46	43.00
PY	PW	83	83	**1112.50****	83	83	1003.00	55	47	70.00
PW	PB	83	83	87.00	83	83	418.00	47	53	78.00
PW	BV	83	83	376.50	83	83	800.00	47	46	26.00
PW	PY	83	83	427.50	83	83	888.00	47	55	50.00

Significant difference is indicated in bold;
** denotes $p < 0.01$; * denotes $p < 0.05$.
